# Supplementary material for: Damage dynamics and the role of chance in the timing of E. coli cell death
Source: Nat Commun. 2023 Apr 18;14:2209. doi: 10.1038/s41467-023-37930-x (PMC10113371; doi:10.1038/s41467-023-37930-x)
Supplement: Supplementary file 4 — Description of Additional Supplementary Files [file 41467_2023_37930_MOESM4_ESM.pdf]

Description of Additional Supplementary Files

File Name: Supplementary Data 1
Description: Kolmogorov-Smirnov (KS) test statistics (one sample) for the 15 distribution functions compared to the marginal damage distributions at different timepoints. Corresponding p-values are shown in Figure S4.

File Name: Supplementary Movie 1
Description: Time-lapse movie of one sample imaging position. Each frame is a pseudo-color image, color-merged from CFP and PI fluorescence images.
