# Supplementary material for: Damage dynamics and the role of chance in the timing of E. coli cell death
Source: Nat Commun. 2023 Apr 18;14:2209. doi: 10.1038/s41467-023-37930-x (PMC10113371; doi:10.1038/s41467-023-37930-x)
Supplement: Supplementary file 6 — Reporting Summary [file 41467_2023_37930_MOESM6_ESM.pdf]

nature portfolio

Corresponding author(s): Yifan Yang, Uri Alon

Last updated by author(s): 19/02/2023

Reporting Summary

Nature Portfolio wishes to improve the reproducibility of the work that we publish. This form provides structure for consistency and transparency in reporting. For further information on Nature Portfolio policies, see our Editorial Policies and the Editorial Policy Checklist.

Statistics

For all statistical analyses, confirm that the following items are present in the figure legend, table legend, main text, or Methods section.

n/a	Confirmed	
☐	☒	The exact sample size (n) for each experimental group/condition, given as a discrete number and unit of measurement
☐	☒	A statement on whether measurements were taken from distinct samples or whether the same sample was measured repeatedly
☐	☒	The statistical test(s) used AND whether they are one- or two-sided<br>*Only common tests should be described solely by name; describe more complex techniques in the Methods section.*
☐	☒	A description of all covariates tested
☐	☒	A description of any assumptions or corrections, such as tests of normality and adjustment for multiple comparisons
☐	☒	A full description of the statistical parameters including central tendency (e.g. means) or other basic estimates (e.g. regression coefficient) AND variation (e.g. standard deviation) or associated estimates of uncertainty (e.g. confidence intervals)
☐	☒	For null hypothesis testing, the test statistic (e.g. F, t, r) with confidence intervals, effect sizes, degrees of freedom and P value noted<br>*Give P values as exact values whenever suitable.*
☒	☐	For Bayesian analysis, information on the choice of priors and Markov chain Monte Carlo settings
☒	☐	For hierarchical and complex designs, identification of the appropriate level for tests and full reporting of outcomes
☐	☒	Estimates of effect sizes (e.g. Cohen's d, Pearson's r), indicating how they were calculated

Our web collection on statistics for biologists contains articles on many of the points above.

Software and code

Policy information about availability of computer code

Data collection	Metamorph version 6 was used to control automatic time-lapse microscopy to collect data.
Data analysis	ImageJ (ImageJ 1.48v, Java 1.6.0_65 32-bit) was used for image analysis. Data processing and statistics was done using Python 3.7 and specifically the scipy.stats package (version 1.7.3). Mathematica 12 was used for analytical derivations and for simulating stochastic differential equations.<br>Custom codes written for analysis, statistics and modeling can be found in the following link: https://github.com/y1fanyang/coliDamageDynamics. Image analysis and longitudinal data can also be found in this link.

For manuscripts utilizing custom algorithms or software that are central to the research but not yet described in published literature, software must be made available to editors and reviewers. We strongly encourage code deposition in a community repository (e.g. GitHub). See the Nature Portfolio guidelines for submitting code & software for further information.

Data

Policy information about availability of data

All manuscripts must include a data availability statement. This statement should provide the following information, where applicable:

- Accession codes, unique identifiers, or web links for publicly available datasets
- A description of any restrictions on data availability
- For clinical datasets or third party data, please ensure that the statement adheres to our policy

Source data are provided with this paper, including the longitudinal time-series which are the main results of our experiments and the primary data that all subsequent analysis and modeling depends on. In addition, all data and statistics underlying Fig. 1d-h, Fig. 2a-e, Fig. 3a-e,g, Fig. 4d-i, Fig. 5e-h, Fig. S4 in are included in the Source Data file. All other data are available from the corresponding authors upon request.

Human research participants

Policy information about studies involving human research participants and Sex and Gender in Research.

Reporting on sex and gender	Not applicable.
Population characteristics	Not applicable.
Recruitment	Not applicable.
Ethics oversight	Not applicable.

Note that full information on the approval of the study protocol must also be provided in the manuscript.

Field-specific reporting

Please select the one below that is the best fit for your research. If you are not sure, read the appropriate sections before making your selection.

☒ Life sciences ☐ Behavioural & social sciences ☐ Ecological, evolutionary & environmental sciences

For a reference copy of the document with all sections, see nature.com/documents/nr-reporting-summary-flat.pdf

Life sciences study design

All studies must disclose on these points even when the disclosure is negative.

Sample size	This study is based on single-cell microscopy experiments, where the sample size is determined by the number of cells recorded in a given experiment. Each experiment recorded ~ 30 imaging positions, and the cells in these positions form the cohort. ΔrpoS cells are morphologically bigger in starvation conditions and thus the same amount of dead end channels contain less cells for ΔrpoS compared to wildtype. Experiments where non enough cells are recorded (<100) are discarded.
Data exclusions	Data gathered from experiments were kept as intact populations, where all individual cells whose trajectories where successfully followed were included in the study.
Replication	The same experiments shown in the paper were repeated both within the same day with 3 duplicate populations, and over multiple days with repeated experiments. Duplicate/repeat cell populations show similar population dynamics (Gompertz law), which is reported in a previous publication. Detailed single-cell trajectories were extracted only for the population shown in the paper, as the image analysis process involved time-consuming manual steps for error corrections.
Randomization	This a population-based longitudinal observational study, where we do not have causal covariates. All individual cells tracked and analyzed are included in the population.
Blinding	Both the microscopy experiments and image analysis were done in a blinded fashion: neither the experimenter nor the analyst performing manual corrections to the automatic segmentation results were aware of the genotype of the cells in question. Genotype labels were only applied to the data after fluorescent timeseries were extracted from the time-lapse images.

Reporting for specific materials, systems and methods

We require information from authors about some types of materials, experimental systems and methods used in many studies. Here, indicate whether each material, system or method listed is relevant to your study. If you are not sure if a list item applies to your research, read the appropriate section before selecting a response.

Materials & experimental systems

n/a	Involved in the study
☒	☐ Antibodies
☒	☐ Eukaryotic cell lines
☒	☐ Palaeontology and archaeology
☒	☐ Animals and other organisms
☒	☐ Clinical data
☒	☐ Dual use research of concern

Methods

n/a	Involved in the study
☒	☐ ChIP-seq
☒	☐ Flow cytometry
☒	☐ MRI-based neuroimaging
